# Real-time multiplex PCR for simultaneous detection of multiple species from environmental DNA: an application on two Japanese medaka species

**DOI:** 10.1038/s41598-018-27434-w

**Published:** 2018-06-14

**Authors:** Satsuki Tsuji, Yuka Iguchi, Naoki Shibata, Iori Teramura, Tadao Kitagawa, Hiroki Yamanaka

**Affiliations:** 1 Graduate School of Science and Technology, Ryukoku University, 1-5 Yokotani, Seta Oe-cho, Otsu, 520-2194 Japan; 2 Program in Environmental Management, Graduate School of Agriculture, Kindai University, 3327-204 Nakamachi, Nara, Nara, 631-8505 Japan; 3 Faculty of Science and Technology, Ryukoku University, 1-5 Yokotani, Seta Oe-cho, Otsu, 520-2194 Japan; 4 The Research Center for Satoyama Studies, Ryukoku University, 1-5 Yokotani, Seta Oe-cho, Otsu, 520-2194 Japan

## Abstract

Information about species distribution is crucial to ecological studies. Environmental DNA (eDNA) analysis has recently been used to estimate the distribution of aquatic organisms. Several analytical methods including metabarcoding and species-specific PCR are being used for eDNA analysis. However, when only a few species are targeted, metabarcoding is not cost-effective because of the wasted consumption of read due to amplification of non-target species DNA. On the other hand, species-specific PCR requires tests to be repeated multiple times resulting in consuming more DNA templates, and experimental consumables. Here we propose a methodological framework for simultaneously detecting a few species using real-time multiplex PCR. We developed the species-specific primer-probe sets for two species of Japanese medaka (*Oryzias latipes* and *o. sakaizumii*), and we used them in the real-time multiplex PCR. In aquarium experiment, even when the species abundances were biased, both species were simultaneously detected in all samples. In a field survey, eDNA analysis and capture survey produced consistent results in all sampling sites, including sites with low fish densities. eDNA analysis using real-time multiplex PCR can be easily applied to other aquatic organisms, enabling a more cost-effective distribution survey of multiple target organisms.

## Introduction

The information on species distributions is crucial not only for basic ecological research but also for population management and conservation. Various surveillance methods such as baited traps, cast nets, and visual observation has conventionally been used for researches on aquatic organisms^1–3^; however, the information obtained from these methods is likely to vary in quality for at least three reasons. First, capture efficiency or discovery rate depends on the researcher’s skill. Second, substantial effort might be needed to find rare species^4^, and this effort can degrade the reliability of “absence” data. Finally, species identification on the basis of morphology usually requires taxonomic expertise^5^. These characteristics of traditional methods will obstruct standardized surveys, particularly large-scale surveys. Furthermore, capture surveys might be unfavorable for endangered species because the sampling activities can damage target-species populations or their habitats. In addition, these traditional methods require a lot of time and labor.

To overcome these obstacles, a technique called “environmental DNA (eDNA) analysis” has recently been used^6,7^. Environmental DNA is a DNA molecule that is released from organisms into the water or soil as components of metabolic waste, damaged tissue, and sloughed skin cells^8^. Environmental DNA analysis is a sensitive and reliable survey method by which species can be detected, especially in aquatic organisms^9–11^. An extremely promising aspect of eDNA analysis is the ability to use the efforts of people who are not trained in the method used to collect samples^12^. Furthermore, eDNA analysis, which relies on genetic information, can readily distinguish species that are morphologically similar to each other^4^. The simplicity of sampling and a well-established laboratory workflow can provide consistent results and minimize any impacts from sampling activity on target-species populations or their habitats. eDNA analysis is also relatively cost-effective. For example, the use of eDNA analysis to determine the distribution of the nearly extinct European weather loach (*Misgurnus fossilis*) decreased the prospective cost of a conventional survey by one-half when they included the investigators’ salaries^13^.

Environmental DNA analysis for detecting aquatic macroorganisms can be divided into two main types, metabarcoding or species-specific PCR^14^. The metabarcoding technique, which uses a next-generation sequencer, enables us to obtain DNA sequence data of phylogenetically broad taxa in a single analysis (e.g., all Teleostei)^15,16^; however, when only a few species are targeted, metabarcoding is not efficient because of the amplification of non-target species DNA. Furthermore, metabarcoding can sometimes fail to distinguish closely related species because of the genetic homology on the amplified DNA region. On the other hand, the species-specific PCR technique, which uses primers that amplify only target-species DNA, is suitable for surveys targeting only a subset of a community (e.g., monitoring an endangered species) and for samples including closely related species that cannot be distinguished by the sequence on the amplified DNA region^6,10^. However, when multiple species are targeted, PCR is required for each individual species^17,18^ so that the cost in time or money and the amount of DNA samples would increase depending on the number of target species.

To address these PCR issues, a new methodological framework is suggested for simultaneous detection of a limited number of species by analyzing eDNA using real-time multiplex PCR. Real-time multiplex PCR uses a set of species-specific primers and probe that is labeled with different fluorescent dyes for each target species so that approximately two to five species (depending on the experimental conditions) can be detected simultaneously in a single real-time PCR reaction. Real-time multiplex PCR, as opposed to real-time single PCR, shortens the processing times and reduces the use of reagents^19,20^. In this study, this methodological framework was applied for detecting the *Oryzias* species complex in Japan, generally called ‘medaka’ fish, which includes *O. latipes* and *O. sakaizumii* (hereafter, *latipes* and *sakaizumii*, respectively). The two medaka species are so morphologically similar that reliable species identification cannot be made without DNA analysis^21^. In addition, they are partially sympatric^22^, yet their distribution on a local scale remains unknown. Both species are listed as ‘Vulnerable’ on the *Red List of Threatened Species of Japan*^23^, and are endangered because of the invasion of non-native species and habitat degradation^24^. The knowledge of species-specific distributions is crucial for their effective management and conservation.

In this study, we developed new species-specific primer probe sets at specific regions of mitochondrial DNA to detect and distinguish the two medaka species in Japan. The specificity of the developed primer probe set for each species was tested using genomic DNA from the respective target species and real-time single PCR. In addition, the developed primer probe sets were tested with respect to whether they could detect only respective target species using real-time multiplex PCR with a mixture of *latipes* and *sakaizumii* genomic DNA. To assess the capability of this detection system, the effect of the biased abundance of the species on the detection capability was examined in aquarium experiment. In addition, the effectiveness of these primer probe sets was tested by determining the distributions of the two medaka species in their known natural habitats.

## Results

No DNA was detected in any experimental control or PCR-NCs, confirming the absence of cross-contamination during sample processing. In *in silico* test using Primer-BLAST (http://www.ncbi.nlm.nih.gov/tools/primer-blast/), the designed primer sets for *latipes* and *sakaizumii* were found to amplified only the target medaka species. In real-time single PCR with extracted genomic DNA, both of the developed primer probe sets for *latipes* and *sakaizumii* showed species specific amplification (Table 1). In real-time multiplex PCR with genomic DNA extracted from single species (*latipes*, *sakaizumii*, mosquitofish, or guppy), both of developed primer probe sets showed species-specific amplification (Table 2). In real-time multiplex PCR with the mixture of extracted genomic DNA from both medaka species, DNA was detected from each (Table 2). In this real-time multiplex PCR with adjusted probe concentrations, each TaqMan probe showed approximately equal fluorescence intensity (*latipes* Rn = 0.97, *sakaizumii* Rn = 0.92).

**Table 1:** Results of the primer-probe specificity test with genomic DNA using real-time single polymerase chain reaction.

Primer probe set	genomic DNA template (0.1 ng)		PCR positive (repeatability)	
			*latipes*	*sakaizumii*
OlaND5-F, R, Pr (real-time single PCR)	*latipes*	1	3/3 (0.14)	—
		2	3/3 (0.20)	—
		3	3/3 (0.11)	—
	*sakaizumii*	1	0/3	—
		2	0/3	—
		3	0/3	—
Osa16S-F, R, Pr (real-time single PCR)	*latipes*	1	—	0/3
		2	—	0/3
		3	—	0/3
	*sakaizumii*	1	—	3/3 (0.19)
		2	—	3/3 (0.03)
		3	—	3/3 (0.13)

The identification numbers of the genomic DNA template indicate different individuals. The repeatability of three PCR replications are shown as SD of Ct values.

**Table 2:** Results of the primer-probe specificity test with genomic DNA using real-time multiplex polymerase chain reaction.

Primer probe set	genomic DNA template (0.1 ng)		PCR positive (repeatability)	
			*latipes*	*sakaizumii*
Both (real-time multiplex PCR)	*latipes*	1	3/3 (0.11)	0/3
		2	3/3 (0.11)	0/3
		3	3/3 (0.17)	0/3
	*sakaizumi*	1	0/3	3/3 (0.10)
		2	0/3	3/3 (0.07)
		3	0/3	3/3 (0.44)
	*latipes* 1 and *sakaizumii* 1		3/3 (0.19)	3/3 (0.14)
	*latipes* 2 and *sakaizumii* 2		3/3 (0.09)	3/3 (0.01)
	*latipes* 3 and *sakaizumii* 3		3/3 (0.22)	3/3 (0.12)
	mosquitofish		0/3	0/3
	guppy		0/3	0/3

The identification numbers of the genomic DNA template indicate different individuals. The repeatability of three PCR replications are shown as SD of Ct values.

In aquarium experiment examining the effect of biased abundance of the two medaka species on the detection capability, both species were simultaneously detected with 50-mL sampling in all replicates of real-time multiplex PCR even when the abundance of the two species was biased.

**Figure 1:**
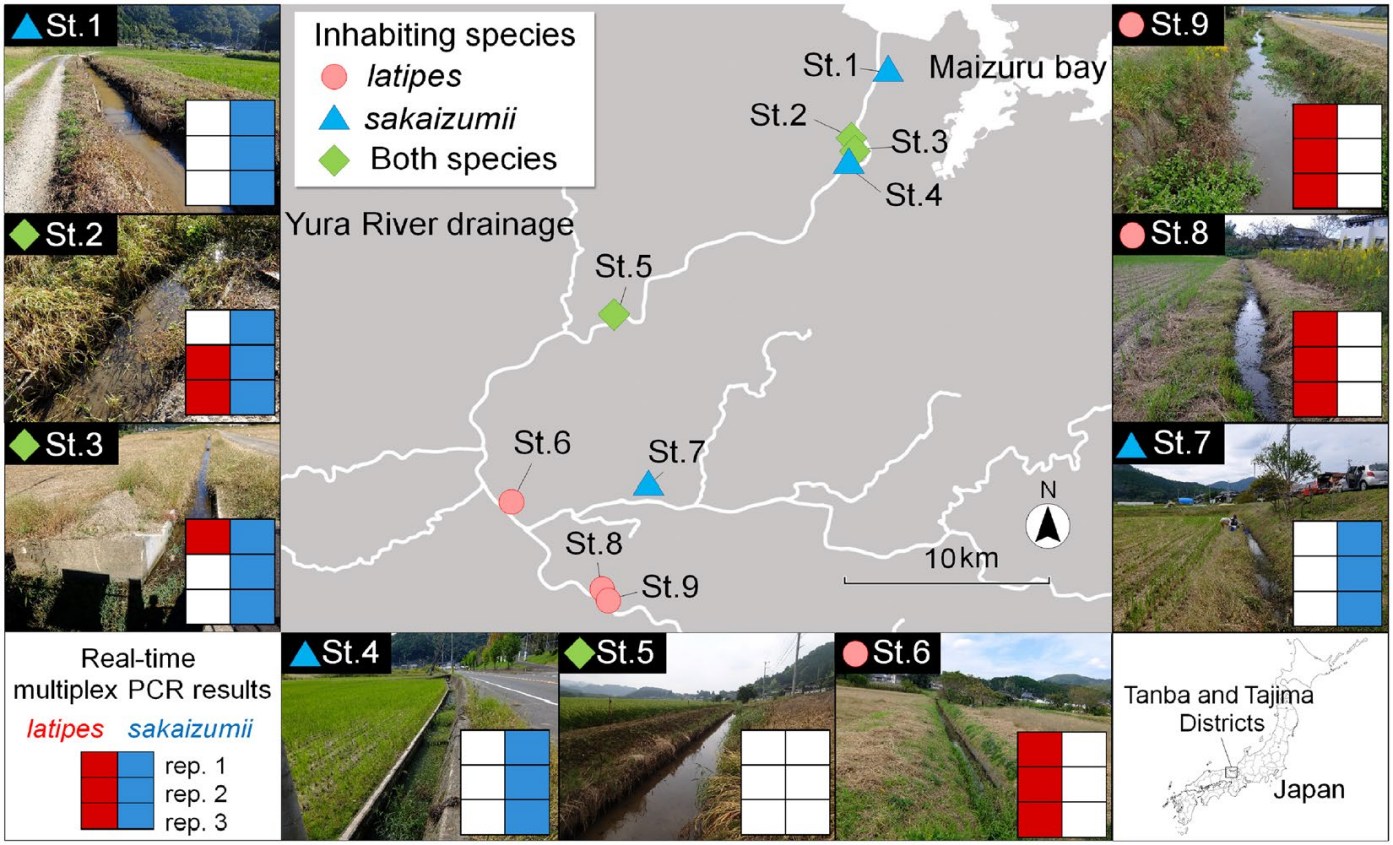
Distribution of the two medaka species determined by the environmental DNA survey with real-time multiplex polymerase chain reaction (PCR) and the capture survey. Results of the capture survey are consolidated data of PCR-restriction fragment length polymorphism analysis in our study and Kume and Hosoya^22^. Marks on each site indicate inhabiting species determined by capture survey (circle; *Oryzias latipes*, triangle; *O. sakaizumii*, diamond; both species). Closed boxes of red (*O. latipes*) and blue (*O. sakaizumii*) indicate the positive results of real-time multiplex PCR with three replications. All photographs were taken by ST. This map was created using QGIS version 2.8 (http://www.qgis.org/en/site/) based on the Administrative Zones Data (http://nlftp.mlit.go.jp/ksj/gml/datalist/KsjTmplt-N03-v2_3.html) and the Rivers Data (http://nlftp.mlit.go.jp/ksj/gml/datalist/KsjTmplt-W05.html) which were obtained from free download service of the National Land Numerical Information (http://nlftp.mlit.go.jp/ksj/index.html, edited by ST). There was no need of obtaining permissions for editing and publishing of map data.

In the field survey, the abundance of medaka species varied among sites, with the calculated CPUE ranging from 0.4 (st. 3) to 100 fish/net*h (st. 8; Table 3). The distributions of the two medaka species inferred by real-time multiplex PCR of eDNA were consistent with those determined in the previous study^22^ and the capture survey (Fig. 1). In the results of PCR-RFLP analysis, both medaka species were found in st. 2 at a ratio of 1 *latipes* to 29 *sakaizumii*, and only *latipes* was found in st. 9. In the analysis of eDNA from st. 2, *latipes* was detected in two out of three PCR replications, whereas *sakaizumii* was detected in all three replications. In st. 9, only *latipes* was detected in all three PCR replications of eDNA samples. In st. 5, no medaka species were captured, and neither species was detected in the eDNA analysis. In the other sites (sts. 1, 3, 4, 6, 7, and 8), the distributions of the two medaka species inferred by our eDNA analysis were consistent with those in the previous report^22^. In the eDNA analysis for these sites, all three PCR replications produced consistent results, but for *latipes* from st. 3, which was detected in only one replication. Amplification specificity of the real-time multiplex PCR of all field-collected samples was confirmed by direct amplicon sequencing (Table S1). In inhibition test, the ΔCt values from the internal controls of all samples were lower than 3, indicating that the inhibition was absence in field-collected samples (Table S2).

**Table 3:** Summary of water quality data, the calculated catch per unit effort, results of reaction polymerase chain reaction–restriction fragment length polymorphism (PCR-RFLP), and reported inhabiting species by Kume & Hosoya^22^ at each sampling site.

St. No.	Water temperature (°C)	pH	Electrical conductivity (mS/cm)	CPUE (fish/net*h)	PCR-RFLP		Reported inhabiting species
					*latipes*	*sakaizumii*	
1 (Yugou)	18	7.87	0.3	3.7	—	—	*sakaizumii*
2 (Kazue)	17.5	8.58	0.35	—	1	29	*sakaizumii*
3 (Maruta)	19.2	8.02	0.35	0.4	—	—	both
4 (Yakumo)	23.2	10.49	0.18	—	—	—	*sakaizumii*
5 (Kita-ariji)	22.9	7.99	0.21	—	—	—	both
6 (Kaminaka)	24.7	8.55	0.2	—	—	—	*latipes*
7 (Kisaichi)	24.9	8.02	0.28	20	—	—	*sakaizumii*
8 (Osada 1)	23.9	7.95	0.14	100	—	—	*latipes*
9 (Osada 2)	22.3	7.99	0.17	20	30	0	—

## Discussion

The new methodological framework using eDNA analysis with real-time multiplex PCR was developed and applied to the two medaka species system in this study. The developed primer probe sets for each medaka species could amplify species specific genomic DNA in both real-time single and multiplex PCR (Tables 1 and 2). When eDNA from the aquariums was analyzed, biased abundance of the two medaka species in an aquarium did not prevent detection of both species in any real-time multiplex PCR with sufficient sample volume. When this detection system was applied in the field survey, the distributions of medaka species inferred by eDNA were consistent with those of capture survey results, including sites with low fish density (Table 3, Fig. 1). Thus, our detection system for the two medaka species is reliable and useful for determining their distributions, and in general, eDNA analysis with real-time multiplex PCR would be applicable to other aquatic systems. Real-time multiplex PCR has two advantages over conventional real-time single PCR. First, whereas single PCR requires an amplification reaction for each single target species, real-time multiplex PCR can simultaneously detect a few target species in a single reaction, thereby reducing reagent use, labor, and time. This cost-efficiency makes it possible to increase the numbers of target species and samples. Second, real-time multiplex PCR can reduce the use of PCR samples. The remaining samples can be stored and used for further analyses^25^.

In the aquarium experiment, the less abundant species were detected in all real-time multiplex PCRs even when the abundance between the two medaka species was biased toward one side. Consistent with the aquarium experiment, the less abundant species was successfully detected in the field surveys (*latipes* in sts. 2 and 3). In both sts. 2 and 3 of the field survey, the fish densities of *latipes* were much lower than that of *sakaizumii*. In st. 2, the ratio of the captured individuals of *latipes* to *sakaizumii* was 1:29. In st. 3, CPUE was smallest in all sampling sites, and the relative ratio of the *latipes* to *sakaizumii* was reported to be 1:4^22^. These results suggest that *latipes* is scarcely distributed in these sites. The eDNA concentration increases with the abundance and/or biomass of organisms^26–29^; therefore, the eDNA concentrations of *latipes* in sts. 2 and 3 were most likely low. Less abundance of eDNA generally raises the probability of PCR dropout (i.e., failure in PCR amplification). One or two positives out of three PCR replicates for *latipes* in sts. 2 and 3 might be caused by less abundance of the species in those sites. Multiple PCR replicates appear to reduce false negatives, even in species with a low density, and are possibly also used as a proxy for biomass as suggested by previous studies on the positive relationship between biomass and the number of positives in the PCR replicates^30^.

Our field survey confirmed a new sympatric habitat (st. 2) and the recent disappearance of both medaka species (st. 5). There is a high possibility that the previous study in st. 2^22^ failed to find *latipes* because of the insufficient number of individuals sampled (n = 10) given that our result indicated that *latipes* is much less abundant than *sakaizumii* (1 vs. 29) in this site. In addition, there is another possibility that *latipes* has dispersed into this site after the previous study because st. 2 is located downstream of st. 3, where *latipes* inhabits, and the sites are connected to each other by a runnel (the distance between two sites is ~560 m). In any case, non-invasive sampling and the high reliability of eDNA analysis can facilitate long-term sustainable surveys, especially for endangered species, such as the two medaka species. The absence of both medaka species in st. 5 indicated by the result of both eDNA analysis and the capture survey suggests the recent extinction of them after the previous survey^22^. In st. 5, lining of channels was changed to three-sides concrete after the previous capture survey (2010). This human activity might cause habitat manipulation because the medaka species are vulnerable to anthropogenic habitat disturbances^31,32^. Our eDNA analysis with high detection capability can be an effective tool for tracing a species habitat changes.

Application of real-time multiplex PCR to other systems requires some technical considerations. First, primer probe sets must be highly species specific to accurately detect only the target species. TaqMan probe chemistry was adopted to improve the specificity of the analysis. Second, each primer probe set must have approximately the same melting temperature (Tm) because any difference in Tm might cause DNA amplification bias. Third, uncompetitive primers and probes must be designed and used for the reactions. When more species are targeted in a single reaction, the possibilities of primer (probe) dimer generation and probe fluorescent competition will be increased. Finally, the length of target sequences must be similar among the target species because the amplification efficiency and the eDNA decay rate in ambient water should be affected by the length of the DNA fragment^33^.

The present study has demonstrated that multiple species can be simultaneously detected using real-time multiplex PCR from eDNA although only a few targeted species were included. This eDNA analysis with real-time multiplex PCR should be useful for large-scale and long-term distribution surveys that focus on a few multiple species and could be easily applied to various combinations of species. Real-time multiplex PCR will be more cost-effective than the conventional single PCR method. This cost effectiveness allows the scale-up of distribution surveys with increasing target species and/or sampling sites. eDNA analysis with real-time multiplex PCR will allow us to conduct more effective surveys and monitoring of natural communities without the time and budget constraints, and that will contribute toward our understanding and the conservation of biodiversity.

## Materials and Methods

### Ethics Statement

There was no need of obtaining permissions for conducting this research, including lab experiments, field samplings of water and fish. In the current laws and guidelines of Japan relating to animal experiments of fish, the long-term foraging experiment and the usage of DNA samples are allowed without any ethical approvals from any authorities.

### Primer probe design

The sequence data of the mitochondrial complete genome for the two species of Japanese medaka (*latipes* and *sakaizumii*) and those of two non-target species [*Gambusia affinis* (mosquitofish) and *Poecilia reticulata* (guppy)] both of which are introduced species and have similar habitat preference with medaka were obtained from the National Center for Biotechnology Information database. All sequence data used for designing the primer set of each medaka species are listed in Table S3. The *latipes* primers were designed on the ND5 gene and named OlaND5-F/R, and the *sakaizumii* primers were designed on the 16S rRNA gene and named Osa16S-F/R (Table 4). The designed primers for each of the two medaka species had the species-specific nucleotide at the 3′ ends. Primer-BLAST with the default settings was used to the primer parameter checks and *in silico* tests. The lengths of the PCR products were 108 and 136 bp for *latipes* and *sakaizumii*, respectively. The TaqMan probes for each species, named OlaND5-Pr for *latipes* and Osa16S-Pr for *sakaizumii* respectively, were designed on the PCR products by each of the species amplification primers (Table 4). For simultaneous detection by real-time PCR, the 5′ end of the *latipes* and *sakaizumii* probes were respectively labeled with different fluorescent dyes (Table 4).

**Table 4:** List of designed primers and TaqMan probes.

Target species	Primer name	Sequence (5′–>3′)
*latipes*	OlaND5-F OlaND5-R OlaND5-Pr	TCTTTACTATAATCCTGGCAGTCCTTATC CTGCTGCTAACTCTTTTTGTTGTTC [JOE]-AATCTAACTGCTCGCAAAGTCCCACGACT-[BHQ] (Amplicon length = 108 bp)
*sakaizumii*	Osa16S-F Osa16S-R Osa16S-Pr	ATCTTCAAGTAGAGGTGACAGACCA AACTCTCTTGATTTCTAGTCATTTGTGTC [FAM]-TGGATAGAAGTTCAGCCTC-[NFQ]-[MGB] (Amplicon length = 136 bp)

Fluorescence excitation spectra are as follows: JOE 529 nm, FAM 495 nm. Fluorescence emission spectra are as follows: JOE 555 nm, FAM 520 nm.

### Primer probe test with genomic DNA

The specificity of the designed primer probe sets was confirmed by real-time single PCR with extracted genomic DNA from three individuals each *latipes* and *sakaizumii*. Real-time single PCR was performed in a 15-µL reaction mixture for each sample using the StepOnePlus Real-Time System (Life Technologies, Foster City, CA, USA) and analyzed using StepOneSoftware v2.3. The reaction mixture contained a set of the primers OlaND5-F/R or Osa16S-F/R at a final concentration of 900 nM each, 125 nM TaqMan probe, and 0.1 ng genomic DNA (*latipes* or *sakaizumii*) in 1 × TaqMan gene expression Master Mix (Life Technologies, Carlsbad, CA, USA). To assess the occurrence of unintended cross-contamination, PCR negative controls (PCR-NCs) comprising ultrapure water instead of genomic DNA were prepared. The PCR thermal conditions were as follows: 2 min at 50 °C, 10 min at 95 °C, and 55 cycles of 15 s at 95 °C and 60 s at 60 °C. Real-time single PCR for each DNA sample and PCR-NCs were performed in triplicate.

### Real-time multiplex PCR with genomic DNA

The two tests of real-time multiplex PCR using OlaND5-F/R/Pr and Osa16S-F/R/Pr were conducted to examine (i) whether each species could be specifically detected and (ii) whether both species could be simultaneously detected. All real-time multiplex PCRs were performed in a 15-µL reaction mixture for each sample using StepOnePlus Real-Time System and analyzed using StepOneSoftware v2.3. In addition, final concentrations of TaqMan probes in the real-time multiplex PCRs were adjusted to equalize the fluorescence intensities as follows: Osa16S-Pr was set to 41.7 nM (one-third of OlaND5-Pr) because the fluorescence intensity of *sakaizumii* (Rn = 2.99) was approximately three times stronger than that of *latipes* (Rn = 1.04). The reaction mixture contained four amplification primers at a final concentration of 900 nM each, TaqMan probes OlaND5-Pr at a final concentration of 125 nM and Osa16S-Pr at a final concentration of 41.7 nM, and 0.1 ng genomic DNA of one of the four species (*latipes*, *sakaizumii*, mosquitofish or guppy) in 1 × PCR TaqMan gene expression Master Mix. For simultaneous detection test of two medaka species in real-time multiplex PCR, the reaction mixture contained four primers at a final concentration of 900 nM each, TaqMan probes OlaND5-Pr at a final concentration of 125 nM, and Osa16S-Pr at a final concentration of 41.7 nM, and a 0.2 ng mixture of genomic DNA from the two medaka species in 1 × PCR TaqMan gene expression Master Mix. The PCR-NC preparation for both tests used ultrapure water instead of genomic DNA. The thermal condition for the real-time multiplex PCR was same as real-time single PCR. All real-time multiplex PCRs for each DNA sample and PCR-NCs were performed in triplicate. All of the aforementioned real-time PCR complied with the MIQE checklist^34^ (Table S4).

### Aquarium experiments with biased abundance

To examine the effect of biased abundance of the two medaka species on detection capability, our detection system was applied to the aquariums with varying abundance ratios for each medaka species. Thirteen aquariums (35 × 20 × 21 cm) that contained 6 L aged tap water were prepared and were aerated throughout the experiment. In 12 of the 13 aquariums, 10 individuals of adult medaka were placed in each aquarium at a varying abundance ratio of *latipes* to *sakaizumii* (1:9, 5:5, and 9:1, with four replications each). An aquarium without a target species was also prepared as the experimental control to check for cross-contamination during the aquarium experiment. The aquariums were kept in the laboratory at 20 °C room temperature with a 12-h light/dark cycles. After maintaining the fish for 4 days with no food, 50 mL surface water was collected from the center of each aquarium. The collected water samples were immediately filtered through glass fiber filters with a mesh size of 0.7 µm (GF/F, GE Healthcare Japan, Tokyo, Japan). Each filter disc was folded inward in half, wrapped in aluminum foil, placed in a plastic bag with a zipper, and stored at −20 °C until DNA extraction. To avoid contamination, all sampling and filtering equipment were dipped in a 10% bleach solution for >5 min, carefully washed with tap water, and finally rinsed with ultrapure water.

Environmental DNA was extracted from the filter discs following the procedures of Yamanaka *et al*.^35^. Each filter disc was rolled into a cylindrical shape and placed into a spin column (EZ-10 SpinColumn & Collection Tube; Bio Basic Inc., Ontario, Canada), from which a silica-gel membrane was prospectively removed. After removing excess water on the filter by centrifugation, the mixture, containing 200 µL ultrapure water, 100 µL buffer AL, and 10 µL proteinase K, was added to the filter. The spin columns were incubated at 56 °C for 15 min. The spin columns were then centrifuged for 1 min at 6000 × *g*, and upper parts of the spin columns were removed and placed on new 2-mL collection tubes. Then, 200 µL TE buffer (pH 8.0) was added onto the filter, which was incubated for 1 min at room temperature. Spin columns were centrifuged for 1 min at 6000 × *g*, and the elution was mixed with the first filtrate, 200 µL buffer AL and 600 µL 100% ethanol. The mixture was then purified using the DNeasy Blood & Tissue Kit (Qiagen, Hilden, Germany) according to the manufacturer’s instructions. At the final elution step, DNA was eluted from the DNeasy spin column with 100 µL of buffer AE. The buffer AL, buffer AE and proteinase K used for DNA extraction were the DNA attachment reagents from the extraction kit. The extracted eDNA was amplified by real-time multiplex PCR in the same manner as described above for real-time multiplex PCR with genomic DNA. All reactions were performed on a single run. As a DNA template, each 1 µL DNA sample was added.

### Assay of field-collected samples

A field survey using our detection system was conducted to examine whether the detection system could be applied for practical researches. This survey was conducted at the drainages of the Yura River system in the Tanba and Tajima Districts, Japan. The eight sampling sites (sts. 1–8) selected were the same sites as those from Kume & Hosoya^22^ with new st. 9 added for this study (Fig. 1). All water sampling was conducted on October 16, 2016. The 0.5 L of surface water was collected using a plastic cup in each sampling site. To avoid contamination, different equipment was used for each water sampling, and different investigators, respectively, performed water sampling and capture survey. The water quality of each sample is shown in Table 3. Collected water samples were filtered immediately using the on-site water filtration system^35^ with GF/F filters and were immediately kept at −20 °C until use. As an experimental control, the same volume of ultrapure water was filtered and treated in the same manner as that in the samples. DNA was extracted from the filters using the same method as that used in the aquarium experiments. The extracted DNA was amplified by real-time multiplex PCR in the same manner as above for real-time multiplex PCR with genomic DNA. Two microliters of DNA samples were used as the DNA template. The PCR amplified products were commercially sequenced using the Sanger sequencing method.

After water sampling, three investigators conducted the capture survey at sts. 1, 3, 5, 7, 8, and 9 using hand nets. The number of captured individuals was limited to a maximum of 30 fish at each sampling site, and they were carefully handled as much as possible to avoid pain and stress. Captured fish were quickly fixed in 70% ethanol onsite. The catch per unit effort (CPUE) as the number of fish/net* hour was calculated and used as the index of fish density. In st. 2, Kume & Hosoya^22^ found only *sakaizumii*, but both medaka species were found in a later survey (Iguchi *et al*. unpublished); therefore, the PCR-restriction fragment length polymorphism (PCR-RFLP) analysis on cytochrome *b* genes was performed using captured individuals from sts. 2 and 9 to determine the resident species. For PCR-RFLP analysis, total genomic DNA was extracted from muscle or fin tissues of the 30 captured individuals using conventional phenol-chloroform methods^36^. For st. 2, the 30 individuals captured on June 20, 2016, were used for PCR-RFLP analysis following Takehana *et al*.^37^. PCR-amplified segments were digested with enzyme *Hae*III, and the fragments were confirmed by electrophoresis on 3% agarose gels, dyed with Midori Green (Nippon Genetics Europe GmbH, Duren, Germany). The two medaka species were distinguished based on the respective diagnostic fragments patterns (pattern A–I for *sakaizumii* and pattern J–Z for *latipes*). Interestingly, Kume & Hosoya^22^ found both medaka species in st. 5, but neither species has been found since 2013 because of the subsequent revetment construction (Iguchi *et al*. unpublished).

### PCR inhibition test for field-collected eDNA samples

To evaluate the presence or absence of PCR inhibition, the Ct shift was compared between the field-collected samples and controls (only DNA elution buffer) with the same number of foreign DNA copies. When PCR inhibition was occurred by PCR inhibitors in field-collected sample, Ct for a given quantity of foreign DNA would shift (delay), comparing with the controls. To check whether the field-collected eDNA samples cause the PCR inhibitions or not, known DNA copies of *Trachurus japonicus* (Japanese jack mackerel), a marine fish and does not inhabit in the study sites, were spiked in the PCR reactions with eDNA sample or Buffer AE, respectively. The primer probe set reported in Yamamoto *et al*.^29^ was used: Tja-CytB-Forward primer, 5′-CAGATATCGCAACCGCCTTT-3′; Tja-CytB-Reverse primer, 5′-CCGATGTGAAGGTAAATGCAAA-3′; Tja-CytB-Probe, 5′-FAM- TATGCACGCCAACGGCGCCT-TAMRA-3′. Each PCR reaction mixture (15 μL total volume) contained 900 nM of each primer (Tja-CytB-F and -R), 125 nM TaqMan probe (Tja-CytB-Pr), 2 μL of the field-collected DNA sample or Buffer AE, and plasmid DNA containing the cytochrome *b* gene of *T. japonicus* (1.5 × 10^2^ copies) in 1 × PCR TaqMan gene expression Master Mix. The real-time PCR was performed in triplicate. Ct values of the PCR results from the two settings were compared and calculated as ΔCt = Ct _sample_ − Ct _control_. When Ct values shift ≥3 cycles, it was considered as the evidence of inhibition^30,38–40^.

## Electronic supplementary material

Supplementary Information
