# Supplementary material for: Real-time multiplex PCR for simultaneous detection of multiple species from environmental DNA: an application on two Japanese medaka species
Source: Sci Rep. 2018 Jun 14;8:9138. doi: 10.1038/s41598-018-27434-w (PMC6002393; doi:10.1038/s41598-018-27434-w)
Supplement: Supplementary file 1 — Supplementary Information [file 41598_2018_27434_MOESM1_ESM.docx]

**Real-time multiplex PCR for simultaneous detection of multiple species**

**from environmental DNA: an application on two Japanese medaka species**

Satsuki Tsuji^1,*^, Yuka Iguchi^2^, Iori Teramura^3^, Naoki Shibata^1^, Tadao Kitagawa^2^, Hiroki Yamanaka^3,4^

^1^ Graduate School of Science and Technology, Ryukoku University, 1-5 Yokotani, Seta Oe-cho, Otsu, 520-2194, Japan

^2^ Program in Environmental Management, Graduate School of Agriculture, Kindai University, Nara, Nara, 631-8505, Japan

^3^ Faculty of Science and Technology, Ryukoku University, 1-5 Yokotani, Seta Oe-cho, Otsu, 520-2194, Japan

^4^ The Research Center for Satoyama Studies, Ryukoku University, 1-5 Yokotani, Seta Oe-cho, Otsu, 520-2194, Japan

* Corresponding author

Satsuki Tsuji

Graduate School of Science and Technology, Ryukoku University, 1-5 Yokotani, Seta Oe-cho, Otsu, 520-2194, Japan

E-mail: satsuki.may425@gmail.com

**Table S1. The sequence data that were obtained from amplicons of field samples.**

| st. No | st. name | species | The determined sequence by Sanger sequencing |
| --- | --- | --- | --- |
| 2 | kazue | *latipes* | CTTGTAATCTAGCTGCTCGCAAAGTCCCACGACTAAGCCCTCGCGTTAATTCTA |
| 3 | maruta | *latipes* | CTTGTAATCTAACTGCTCGCAAAGTCCCACGACTAAGCCCTCGCGTTAATTCTA |
| 6 | kaminaka | *latipes* | CTTGTAATCTAACTGCTCGCAAAGTCCCACGACTAAGCCCTCGCGTTAATTCTA |
| 8 | osada1 | *latipes* | CTTGTAATCTAACTGCTCGCAAAGTCCCACGACTAAGCCCTCGCGTTAATTCTA |
| 9 | osada2 | *latipes* | CTTGTAATCTAACTGCTCGCAAAGTCCCACGACTAAGCCCTCGCGTTAATTCTA |
| 1 | yugo | *sakaizumii* | ATCGAACTAAGTTATAGCTGGTTGCTCGTGAAATGGATAGAAGTTCAGCCTCTTGCTTTCTAAATTTTAGCCTAGCATTCTT |
| 2 | kazue | *sakaizumii* | ATCGAACTAAGTTATAGCTGGTTGCTCGTGAAATGGATAGAAGTTCAGCCTCTTGCTTTCTAAATTTTAGCCTAGCATTCTT |
| 3 | maruta | *sakaizumii* | ATCGAACTAAGTTATAGCTGGTTGCTCGTGAAATGGATAGAAGTTCAGCCTCTTGCTTTCTAAATTTTAGCCTAGCATTCTT |
| 4 | yakumo | *sakaizumii* | ATCGAACTAAGTTATAGCTGGTTGCTCGTGAAATGGATAGAAGTTCAGCCTCTTGCTTTCTAAATTTTAGCCTAGCATTCTT |
| 7 | kisaichi | *sakaizumii* | ATCGAACTAAGTTATAGCTGGTTGCTCGTGAAATGGATAGAAGTTCAGCCTCTTGCTTTCTAAATTTTAGCCTAGCATTCTT |

**Table S2. Results of inhibition test using Trachurus japonicus DNA as internal positive control.**

| st. No | Ct | ΔCt (Ct _sample_ – Ct _control_) |
| --- | --- | --- |
| 1 | 32.24 | 1.51 |
| 2 | 33.58 | 0.17 |
| 3 | 33.90 | -0.15 |
| 4 | 32.66 | 1.09 |
| 5 | 33.94 | -0.19 |
| 6 | 33.37 | 0.38 |
| 7 | 33.39 | 0.36 |
| 8 | 33.09 | 0.66 |
| 9 | 33.60 | 0.15 |
| control | 33.75 |  |

**Table S3. A list of all sequence data used for designing the primer set of each medaka species.**

| Species | Accession number | Sequence name |
| --- | --- | --- |
| *Oryzias latipes* | AP008946 | Oryzias_latipes_mitochondrial_DNA_complete_genome_isolate:_Nago |
|  | AP008938 | Oryzias_latipes_mitochondrial_DNA_complete_genome_isolate:_HdrR |
|  | AP008944 | Oryzias_latipes_mitochondrial_DNA_complete_genome_isolate:_Amino |
|  | AB498065 | Oryzias_latipes_mitochondrial_DNA_nearly_complete_genome_strain:_Hd-rR |
|  | AP004421 | Oryzias_latipes_mitochondrial_DNA_complete_genome |
|  | AP008945 | Oryzias_latipes_mitochondrial_DNA_complete_genome_isolate:_Matsuyama |
| *Oryzias sakaizumii* | AP008939 | Oryzias_latipes_mitochondrial_DNA_complete_genome_isolate:_HNI |
|  | AB498066 | Oryzias_latipes_mitochondrial_DNA_nearly_complete_genome_strain:_HNI |
|  | AP008942 | Oryzias_latipes_mitochondrial_DNA_complete_genome_isolate:_Niigata |
|  | AP008941 | Oryzias_latipes_mitochondrial_DNA_complete_genome_isolate:_Hirosaki |
|  | AP008940 | Oryzias_latipes_mitochondrial_DNA_complete_genome_isolate:_Kaga |
|  | AP008943 | Oryzias_latipes_mitochondrial_DNA_complete_genome_isolate:_Toyooka |
| *Poecilia reticulata* (guppy) | KJ013505 | Poecilia_reticulata_mitochondrion_complete_genome |
|  | AB898687 | Poecilia_reticulata_mitochondrial_DNA_complete_genome |
|  | KJ460033 | Poecilia_reticulata_mitochondrion_complete_genome |
|  | NC_024238 | Poecilia_reticulata_mitochondrion_complete_genome |
| *Gambusia affinis* (mosquitofish) | AP004422 | Gambusia_affinis_mitochondrial_DNA_complete_genome |

**Table S4. MIQE Guidelines Checklist.**

| **ITEM TO CHECK** | **IMPORTANCE** | **CHECKLIST** |
| --- | --- | --- |
| **EXPERIMENTAL DESIGN** |  |  |
| Definition of experimental and control groups | **E** | Aquarium experiment Experimental groups: Total 10 fishes were maintained. Control groups: No fish was maintained. |
| Number within each group | **E** | Aquarium experiment Experimental groups: 12 Control groups:1(to check for cross-contamination) |
| Assay carried out by core lab or investigator's lab? | D | Investigator's Lab |
| Acknowledgement of authors' contributions | D | Yes |
| **SAMPLE** |  |  |
| Description | **E** | Aquarium experiment for examine the effect of biased abundance of the two medaka species on detection capability. Field survey to examine whether the developed detection system could be applied for practical researches. |
| Volume/mass of sample processed | D | Aquarium experiment: 50 mL of surface water from the center of each aquarium. Field survey: 0.5 L of surface water from each sampling site (Yura River system). |
| Microdissection or macrodissection | **E** | N/A |
| Processing procedure | **E** | Water sample was ﬁltered using GF/F glass filters (mesh size: ~0.7 μm, GE Healthcare Japan, Tokyo, Japan), and the filters were stored at –20 °C until DNA extraction. The captured DNA was collected from each filter by centrifuge using spin column (EZ-10 SpinColumn & Collection Tube; Bio Basic Inc., Ontario, Canada) and extracted with the DNeasy Blood & Tissue Kit (Qiagen, Hilden, Germany) following the method of Yamanaka et al. (2016). At the final elution step, DNA was eluted from the DNeasy spin column with 100 µL of buffer AE and stored at –20 °C until PCR. |
| If frozen - how and how quickly? | **E** | N/A |
| If fixed - with what, how quickly? | **E** | N/A |
| Sample storage conditions and duration (especially for FFPE samples) | **E** | All samples were stored in 1.5-mL microtubes at –20°C. |
| **NUCLEIC ACID EXTRACTION** |  |  |
| Procedure and/or instrumentation | **E** | We used DNeasy blood and tissue kit (Qiagen, Hilden, Germany). |
| Name of kit and details of any modifications | **E** | We used DNeasy blood and tissue kit following the manufacturer's instructions. |
| Source of additional reagents used | D | N/A |
| Details of DNase or RNAse treatment | **E** | N/A |
| Contamination assessment (DNA or RNA) | **E** | N/A |
| Nucleic acid quantification | **E** | N/A |
| Instrument and method | **E** |  |
| Purity (A260/A280) | D |  |
| Yield | D |  |
| RNA integrity method/instrument | **E** | N/A |
| RIN/RQI or Cq of 3' and 5' transcripts | **E** | N/A |
| Electrophoresis traces | D | N/A |
| Inhibition testing (Cq dilutions, spike or other) | **E** | Not Checked |
| **REVERSE TRANSCRIPTION** |  |  |
| Complete reaction conditions | **E** | N/A |
| Amount of RNA and reaction volume | **E** | N/A |
| Priming oligonucleotide (if using GSP) and concentration | **E** | N/A |
| Reverse transcriptase and concentration | **E** | N/A |
| Temperature and time | **E** | N/A |
| Manufacturer of reagents and catalogue numbers | D | N/A |
| Cqs with and without RT | D | N/A |
| Storage conditions of cDNA | D | N/A |
| **qPCR TARGET INFORMATION** |  |  |
| Gene symbol | **E** | mitochondrial DNA, ND5 (for *latipes*), 16S (for *sakaizumii*) |
| Sequence accession number | **E** | Accession number is listed Table S1. |
| Location of amplicon | D | mitochondrial DNA, ND5 (for latipes), 16S (for sakaizumii) |
| Amplicon length | **E** | 108 bp (for *latipes*), 136 bp (for *sakaizumii*) |
| *In silico* specificity screen (BLAST, etc) | **E** | The Primer-BLAST was used to check the primer parameters and perform *in silico* test. As a result, designed two primer sets amplified only each of target medaka species, respectively. |
| Pseudogenes, retropseudogenes or other homologs? | D | Not Found |
| Sequence alignment | D | N/A |
| Secondary structure analysis of amplicon | D | N/A |
| Location of each primer by exon or intron (if applicable) | **E** | N/A |
| What splice variants are targeted? | **E** | N/A |
| **qPCR OLIGONUCLEOTIDES** |  |  |
| Primer sequences | **E** | OlaND5-F (5’-TCTTTACTATAATCCTGGCAGTCCTTATC-3’) OlaND5-R (5’-CTGCTGCTAACTCTTTTTGTTGTTC-3’) Osa16S-F (5’-ATCTTCAAGTAGAGGTGACAGACCA-3’) Osa16S-R (5’-AACTCTCTTGATTTCTAGTCATTTGTGTC-3’) |
| RTPrimerDB Identification Number | D | Not Submitted |
| Probe sequences | D | OlaND5-Pr (5’-[JOE]-AATCTAACTGCTCGCAAAGTCCCACGACT-[BHQ]-3’) Osa16S-Pr (5’-[FAM]-TGGATAGAAGTTCAGCCTC-[NFQ]-[MGB]-3’) |
| Location and identity of any modifications | **E** | There is no modifications. |
| Manufacturer of oligonucleotides | D | Eurofin |
| Purification method | D | OPC |
| **qPCR PROTOCOL** |  |  |
| Complete reaction conditions | **E** | We indicated complete reaction conditions in manuscript (see "real-time multiplex PCR with genomic DNA"). |
| Reaction volume and amount of cDNA/DNA | **E** | Reaction volume was 15 μL in all experiments. Amount of DNA was different for each experiment, and we indicated it in manuscript. |
| Primer, (probe), Mg++ and dNTP concentrations | **E** | For simultaneous detection test of two medaka species in real-time multiplex PCR, the reaction mixture contained four primers at a final concentration of 900 nM each, TaqMan probes OlaND5-Pr at a final concentration of 125 nM, and Osa16S-Pr at a final concentration of 41.7 nM in 1 × PCR Master Mix (TaqMan gene expression Master Mix; Life Technologies, Carlsbad, CA, USA).　The concentrations of Mg++ and dNTP in TaqMan gene expression Master Mix is undisclosed. |
| Polymerase identity and concentration | **E** | We used TaqMan gene expression Master Mix following the manufacturer's instructions. |
| Buffer/kit identity and manufacturer | **E** | We used TaqMan gene expression Master Mix following the manufacturer's instructions. |
| Exact chemical constitution of the buffer | D | N/A |
| Additives (SYBR Green I, DMSO, etc.) | **E** | N/A |
| Manufacturer of plates/tubes and catalog number | D | MicroAmp Fast 96-well Reaction Plate (0.1 mL, Thermo Fisher Scientific, applied biosystems, MA USA, catalog number; 4346907) & MicroAmp Optical Adhesive Film (Thermo Fisher Scientific, applied biosystems, MA USA, catalog number;4311971) |
| Complete thermocycling parameters | **E** | 2 min at 50 ºC, 10 min at 95 ºC, and 55 cycles of 15 s at 95 ºC and 60 s at 60 ºC |
| Reaction setup (manual/robotic) | D | manual |
| Manufacturer of qPCR instrument | **E** | StepOnePlus Real-Time System (Life Technologies, Foster City, CA, USA) |
| **qPCR VALIDATION** |  |  |
| Evidence of optimisation (from gradients) | D | N/A |
| Specificity (gel, sequence, melt, or digest) | **E** | We checked the sequence of amplicon by direct sequencing (Table S1). |
| For SYBR Green I, Cq of the NTC | **E** | N/A |
| Standard curves with slope and y-intercept | **E** | N/A |
| PCR efficiency calculated from slope | **E** | N/A |
| Confidence interval for PCR efficiency or standard error | D | N/A |
| r2 of standard curve | **E** | N/A |
| Linear dynamic range | **E** | N/A |
| Cq variation at lower limit | **E** | N/A |
| Confidence intervals throughout range | D | N/A |
| Evidence for limit of detection | **E** | N/A |
| If multiplex, efficiency and LOD of each assay. | **E** | N/A |
| **DATA ANALYSIS** |  |  |
| qPCR analysis program (source, version) | **E** | StepOneSoftware v2.3 |
| Cq method determination | **E** | We performed the analysis with default setting of Software above. |
| Outlier identification and disposition | **E** |  |
| Results of NTCs | **E** | All NTCs were PCR negative. |
| Justification of number and choice of reference genes | **E** | N/A |
| Description of normalisation method | **E** | N/A |
| Number and concordance of biological replicates | D | N/A |
| Number and stage (RT or qPCR) of technical replicates | **E** | We performed in three replicates for each qPCR. |
| Repeatability (intra-assay variation) | **E** | Standard deviation (SD) of Ct value among replicates were showed in Table 2 and 3. |
| Reproducibility (inter-assay variation, %CV) | D | N/A |
| Power analysis | D | N/A |
| Statistical methods for result significance | **E** | We interpreted the results based only on PCR positive/negative reaction. |
| Software (source, version) | **E** | StepOneSoftware v2.3 |
| Cq or raw data submission using RDML | D | Not Submitted |
